# Co-producing an assessment instrument for physical activity policies in the early childhood education and care setting

**DOI:** 10.1186/s12966-026-02001-z

**Published:** 2026-10-22

**Authors:** Johannes Brandl, Sylke Oberwöhrmann, Jonas Finger, Maike Till, Stefanie Do, Peter Gelius, Antje Hebestreit, Sven Messing

**Affiliations:** 1 Department of Sport Science and Sport, Friedrich-Alexander-Universität Erlangen-Nürnberg, Gebbertstraße 123B, Erlangen, 91058 Germany; 2 Senate Department for Higher Education and Research, Health, and Long-Term Care, Berlin, Germany; 3 Leibniz-Institute for Prevention Research and Epidemiology - BIPS, Bremen, Germany; 4 Institute of Sport Sciences, University of Lausanne, Lausanne, Switzerland; 5 Physical Activity for Health Research Centre, Health Research Institute, Department of Physical Education and Sport Science, University of Limerick, Limerick, Ireland

**Keywords:** Physical activity, Policy evaluation, Policy assessment, Co-production, Tool development, Childcare, Preschool, Kindergarten

## Abstract

**Background:**

Despite the importance of physical activity (PA) for the physical, cognitive, and socio-emotional development of children, the effects of public policies on PA outcomes in early childhood education and care have only recently been investigated. As the available evidence is relatively recent, it has not yet been integrated into existing policy assessment tools. To address this gap, this study aimed to develop an instrument to assess the PA-related content of policies in early childhood education and care.

**Methods:**

The instrument was developed through a multi-phase co-production process involving researchers and representatives from state-level health reporting authorities in Germany. Development was guided by a conceptual framework for policy content analysis and comprised three phases: (1) prototype development based on a systematic analysis of all 16 German educational framework plans, a systematic literature review on the effectiveness of public policies promoting PA in early childhood education and care, and a structured search for national and international policy recommendations; (2) expert consultation using a two-round Delphi survey (*n* = 24 in round 1; *n* = 26 in round 2), with indicators rated on a 10-point Likert scale and summarized using median scores; and (3) pilot testing in five German federal states to assess feasibility, clarity, and applicability of the instrument.

**Results:**

The initial prototype of the assessment instrument comprised 24 indicators. Several revisions throughout the development process resulted in a final version containing 28 indicators across eight thematic domains (e.g., pedagogical concept, education-related objectives, outdoor PA opportunities, indoor PA opportunities). Pilot testing confirmed the instrument’s applicability and feasibility within state-level health reporting, and minor refinements were made to improve the clarity and completeness of individual indicators. Time needed to complete the instrument ranged from approximately one to six hours.

**Conclusions:**

This study developed the first instrument specifically designed to assess the PA-related content of early childhood education and care policies. This may support intersectoral collaboration between the health and youth sector, and could inform future decision-making processes. Moreover, future studies may test its applicability in other countries.

**Supplementary Information:**

The online version contains supplementary material available at https://doi.org/10.1186/s12966-026-02001-z .

## Background

Physical activity (PA) in early childhood is essential for overall development, with benefits not just for physical health (e.g., reduced obesity, better bone and skeletal health, improved cardiometabolic and motor outcomes) but also for cognitive and socio-emotional functioning [1–3]. Evidence-based recommendations emphasize that PA promotion should begin in early childhood [4–6], as shaping PA behaviors already early in life is associated with lifelong activity [7]. In 2019, the World Health Organization (WHO) published its first global guidelines for children under the age of 5 years, recommending a daily minimum of 180 min of PA by combining active play in childcare and home settings [4]. A recent meta-analysis of 48 studies measuring PA objectively has shown that 60% of preschool-aged children meet these recommendations, with a significantly smaller proportion in girls [8]. Other studies identified sociocultural and socioeconomic determinants as factors influencing the PA levels of preschool-aged children [9, 10].

Furthermore, WHO’s Global Action Plan on Physical Activity (GAPPA) highlights the necessity of multisectoral policy action and specifically advocates for strengthening PA opportunities in early childhood settings such as preschool and childcare [11]. Similarly, UNESCO has developed policy guidelines for the provision of Quality Physical Education in educational settings, including early years, aiming to contribute to the voluntary global target for a 10% relative reduction in the prevalence of insufficient PA [12]. However, these global policy documents provide limited guidance on specific measures for the promotion of PA in the early childhood education and care (ECEC) setting, which has been defined as educational or developmental programs and childcare services for children from birth to the start of compulsory primary education [13]. Further, the term PA promotion covers all measures that directly or indirectly influence the PA behavior at population level, including but not limited to physical education and the development of PA-related competences.

Many studies have investigated the effectiveness of interventions to promote PA in the ECEC setting [14–17], and several instruments have been developed to assess PA-related environments or standards. Examples include the Environment and Policy Assessment and Observation (EPAO) instrument that examines environments, practices, and policies influencing nutrition and PA within ECEC settings [18] and the Go Nutrition and Physical Activity Self-Assessment for Child Care (Go NAPSACC), which supports ECEC providers in evaluating and improving practices and environmental conditions within their facilities [19]. In addition, the ‘Achieving a State of Healthy Weight’ report analyzes to what extent licensing regulations in the United States incorporate or support evidence-based obesity prevention standards for ECEC programs [20]. Even though some of these instruments assess organizational policies or specific types of regulations, they do not assess the PA-related content of public policies in the ECEC sector in a comprehensive way. Only recently, the effects of public policies – defined as “the set of activities that governments engage in for the purpose of changing their economy and society” [21] – on PA promotion in the ECEC setting have been investigated more comprehensively [22–24]. This research has shown, for instance, that public policies can have positive effects by including PA in the curriculum, requiring a minimum of 60 minutes PA per day or revising accreditation standards [23].

In the field of PA promotion, different instruments have been developed to monitor or assess public policies [25, 26]. Prominent examples include WHO’s monitoring framework for the Global Action Plan, the European Union’s Health-Enhancing Physical Activity Monitoring Framework, and research-driven approaches such as the Active Healthy Kids Report Cards [27–29]. While most of these assessment instruments and approaches provide useful information for school-aged children and adolescents, they usually do not cover public policies in the ECEC setting [30]. WHO’s GAPPA Monitoring Framework is a rare example for an instrument which collects data on this aspect; however, the framework only assesses whether a country has a policy promoting PA in childcare settings. To the best of our knowledge, there is no instrument which allows assessing the content of PA policies in the ECEC setting from a PA promotion perspective.

To address this gap, an instrument was developed to assess the PA-related content of public policies in the ECEC setting. The instrument was developed in the German context, where the ECEC setting refers to children from birth up to primary education, which typically corresponds to an age range of 0–6 years [31]. While the primary purpose of the instrument is to inform future data collections in Germany across all sixteen federal states, this study might also be of added value for researchers, policymakers and practitioners in other countries, as it answers the research question: Which indicators are relevant to assess the content of policies for the ECEC setting from a PA promotion perspective?

## Methods

### Study design

The instrument’s development was based on a sequential study design, including three phases: (1) prototype development based on a systematic literature review, an analysis of ECEC policy documents, and a review of policy recommendations; (2) expert consultation via a two-stage Delphi process and additional expert input; and (3) pilot testing by federal health reporting departments.

The study utilized a co-production process [32] involving a research team compiled of six academics from several institutions (JB, MT, SD, PG, AH, SM) and two government officials from the technical level of the Berlin Senate Department for Higher Education and Research, Health and Long-Term Care (SO, JF). Monthly project meetings from May 2023 until November 2025 allowed continuous exchange within the research team and ensured that both academic and government representatives contributed to key decisions (e.g., study design, selection and refinement of indicators). To enhance the applicability and uptake of results in the German political administration, government officials from four additional federal states (Brandenburg, Schleswig-Holstein, North Rhine-Westphalia, Thuringia) advised the project approximately twice per year by consulting strategic decisions of the research team and providing feedback on prototypes of the assessment instrument.

### Study context

This study was conducted in the context of the German federal health reporting (*Gesundheitsberichterstattung*), which is a key instrument for monitoring population health and informing policy-making at different levels of government [33]. In 2021, a state level working group of the Supreme Health Authorities adopted a prevention indicator system for the German federal states which guides the monitoring of conditions, determinants and health outcomes relevant for prevention policies [34, 35]. This prevention indicator system also aims to assess key state-level ECEC policies from a PA promotion perspective, which provided the formal mandate and rationale for developing this instrument. Intended users of this instrument are the representatives of state level health reporting authorities, i.e., government officials within the health sector. However, the new instrument aims to support the analysis of the PA-related content of ECEC policies. In the German context, educational framework plans issued by the state-level youth policy sector were considered as primary policy documents and therefore selected for assessment. Those framework plans exist for each of the 16 German federal states and describe how the legally mandated educational and caregiving responsibilities in ECEC settings should be implemented in practice. They serve as non‑binding guidance rather than curricula, outlining pedagogical principles, developmental goals, and key learning areas for children [36].

### Conceptual framework

**Fig. 1:**
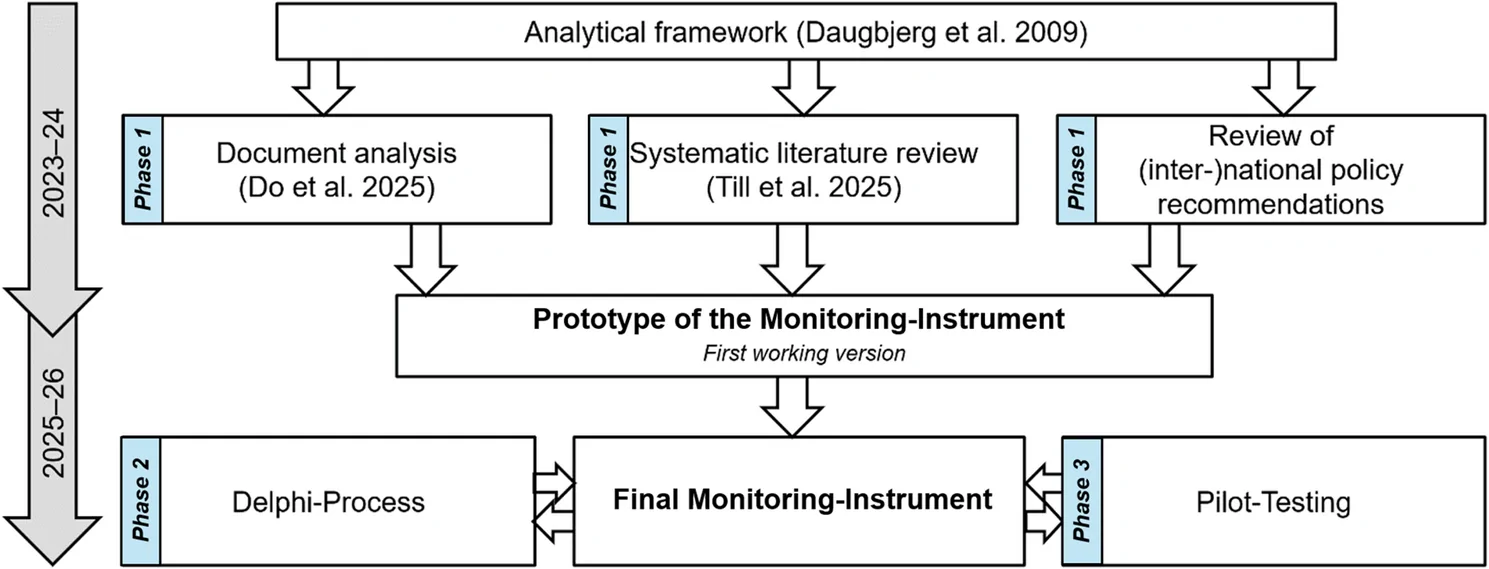
Flowchart showing the development process and corresponding timeline

The development process (Fig. 1) comprised three consecutive phases: prototype development, expert consultation, and pilot testing. It was guided by Daugbjerg et al.’s [37] content analysis grid for PA policy documents, which was used as a conceptual framework in the initial phase of this study. This analysis grid includes eight key criteria intended to support a standardized analysis and comparisons between policy documents based on a set of indicators of good practice for policy development: (1) multisectoral involvement, (2) clarity of implementation responsibilities, (3) legal status, (4) defined target groups, (5) measurable goals, (6) timeframe for policy implementation, (7) allocated budgets, and (8) provisions for evaluation and monitoring. These criteria were used as categories during the development of the initial version of the instrument in phase 1.

### Phase 1 – prototype development

The first phase of instrument development focused on establishing an evidence-informed foundation by combining analyses of existing policy documents, scientific literature, and policy recommendations.

As a starting point, the educational framework plans of all 16 German federal states were systematically collected and analyzed. The content analysis was guided by ten predefined criteria: the eight Daugbjerg criteria and two criteria based on the terminology used in the German prevention indicator system: (9) the presence of PA as a topic in the policy document, and (10) the availability of a pedagogical concept for PA. The latter two criteria reflect two substantive elements specified in the above-mentioned prevention indicator system for the German federal states, which requires PA to be anchored in the educational framework plan and linked to a concrete pedagogical concept. For each educational framework plan, PA-related content was screened using standardized search terms (e.g., “physical activity,” “sport,” “exercise”). Information was extracted independently by two researchers, with discrepancies resolved through discussion. This excerpt was complemented by information provided from the government officials in the youth sector, with one designated contact person from each state’s responsible department for this policy. This information was collected via a brief survey aimed at obtaining additional policy documents relevant for the educational framework plan of the respective federal state, information on its legal status, and the extent of its implementation and evaluation [38].

In addition, a systematic literature review on the effectiveness of public policies promoting PA in the ECEC setting was conducted [23]. For this review, we searched nine electronic databases in August 2023 for studies examining governmental or agency policies affecting PA environments or behaviors in ECEC. We extracted both quantitative and qualitative outcomes, performed a formal quality assessment using the Downs and Black checklist, and summarized study results using a narrative synthesis. The details of this review have been published elsewhere [23].

Furthermore, a structured web search was performed using the Google search engine to identify national and international policy recommendations for PA promotion in ECEC. Search terms combined variations of the keywords “*physical activity*”, “*childcare*” (including “early childhood”, “kindergarten”, and “day care”), and “*recommendations*” or “*guidelines*”. Results for each combination of terms were screened, and documents were included if they provided recommendations on how ECEC institutions can promote PA. Documents merely specifying individual-level recommendations – such as quantitative PA guidelines recommending daily minutes of activity – were excluded if they did not include policy recommendations for promoting PA. Potentially relevant documents were screened by two researchers, and decisions were reached through consensus. Data extraction included the names of the documents, the use of theoretical concepts, aims, implementation guidance, and evaluation criteria.

Collectively, the findings of these analyses were synthesized into a first set of indicators and served as the basis for a prototype of the instrument (Fig. 1).

### Phase 2 – expert consultation

The prototype of the instrument was refined and evaluated using a two-round Delphi survey. Based on available methodological guidance for Delphi studies [39, 40] and an approach used for the development of another PA policy assessment instrument [41], a questionnaire was developed and approved by the partners from academia and political administration. Experts from Germany were invited to assess the importance of the assessment instrument’s indicators; these experts were recruited based on their expertise in (a) PA promotion in early childhood, (b) kindergarten practice, or (c) a combination of both.

In total, 70 experts from different regions of Germany and different backgrounds (academia, policy, practice) were contacted via email for each Delphi round and invited to participate. In round 1 of the online survey, 24 participants (academia: *n* = 9, policy: *n* = 7, practice: *n* = 8) provided qualitative feedback on the clarity, relevance, and completeness of instrument indicators (response rate = 34%). Based on this input, indicators were revised, merged, or newly developed through internal agreement within the project team. In round 2, 26 experts (academia: *n* = 14, policy: *n* = 8, practice: *n* = 4) responded to the Delphi survey (response rate = 37%). Here, the adapted indicators were subjected to quantitative rating on a 10-point Likert scale (1 = “not relevant”, 10 = “extremely relevant”). Median scores were calculated for each indicator to summarize the experts’ assessment of their relevance. These median ratings were used to describe the level of expert agreement and did not serve as a predefined exclusion criterion.

### Phase 3 – pilot testing

As a final step, a pilot test was conducted to evaluate whether the instrument was suitable for application within the German federal health reporting system by its intended end users. The instrument was therefore piloted in the five German states involved in the project (Berlin, Brandenburg, Schleswig-Holstein, North Rhine-Westphalia, Thuringia). To support the pilot test, a manual was developed to guide users through the assessment process and to provide good practice examples from Germany for each indicator. These examples were identified by two reviewers who screened all 16 educational framework plans, using predefined search terms aligned with the content of each indicator. Potentially relevant examples were selected based on their ability to showcase how thematic domains and corresponding indicators can be documented in practice.

The intended users of the assessment instrument – government officials from the health reporting departments – applied the refined prototype to the educational framework plan of their own state under routine working conditions and within realistic time constraints to evaluate the feasibility of future implementation in regular health reporting. The assessment followed a domain-based scoring approach – guided by the manual and the good practice examples – in which each indicator was rated dichotomously (“yes”/“no”) to maximize reproducibility and comparability across federal states. Affirmative responses were scored as one point and summarized for each federal state. For the analysis, responses were aggregated across the participating states to determine the total number of states fulfilling each indicator, expressed as percentage values.

Qualitative feedback was collected from five government officials from the health sector in a single two-hour online meeting. The discussion was guided by predefined questions on feasibility, clarity, and applicability of both the instrument and the manual (e.g., time required to complete the instrument, comprehensibility of individual indicators, usefulness of selected practice examples). Answers and comments were documented and synthesized (i.e., grouping of thematically similar feedback according to recurring themes) by the project team, informing final revisions to the wording, structure, and scoring approach of the assessment instrument.

## Results

### Prototype and Delphi survey

The prototype of the instrument (phase 1) comprised 24 indicators and was informed by (a) an analysis of all 16 federal educational framework plans in Germany [38], (b) eleven studies from the United States, Canada, and Australia, synthesized through a systematic review on the effectiveness of public policies for PA promotion in ECEC [23], and (c) policy recommendations on PA promotion in ECEC from three international [42–44] and three national sources [45–47].

In phase 2, the structure and content of the instrument were adapted based on (a) the Delphi survey, (b) an expert consultation, and (c) an additional policy document providing guidance on PA promotion in the ECEC setting [48]. After the first Delphi round, 16 indicators were reworded, 5 new indicators were added, 2 indicators were merged, and 2 indicators were removed. In the second Delphi round, expert consensus on the relevance of individual indicators was evaluated using median scores, with 26 out of 28 indicators receiving high ratings (≥ 8/10). At the end of phase 2, the project team excluded several groups of indicators from the instrument due to the limited feasibility of data collection. As only a document analysis of educational framework plans was deemed feasible for the intended users from the health sector, aspects that are typically not regulated within these policy documents were excluded (e.g., legal status, financial resources, size specifications for indoor and outdoor areas, and evaluation mechanisms). The second phase resulted in a version containing 29 indicators.

### Pilot test

**Fig. 2:**
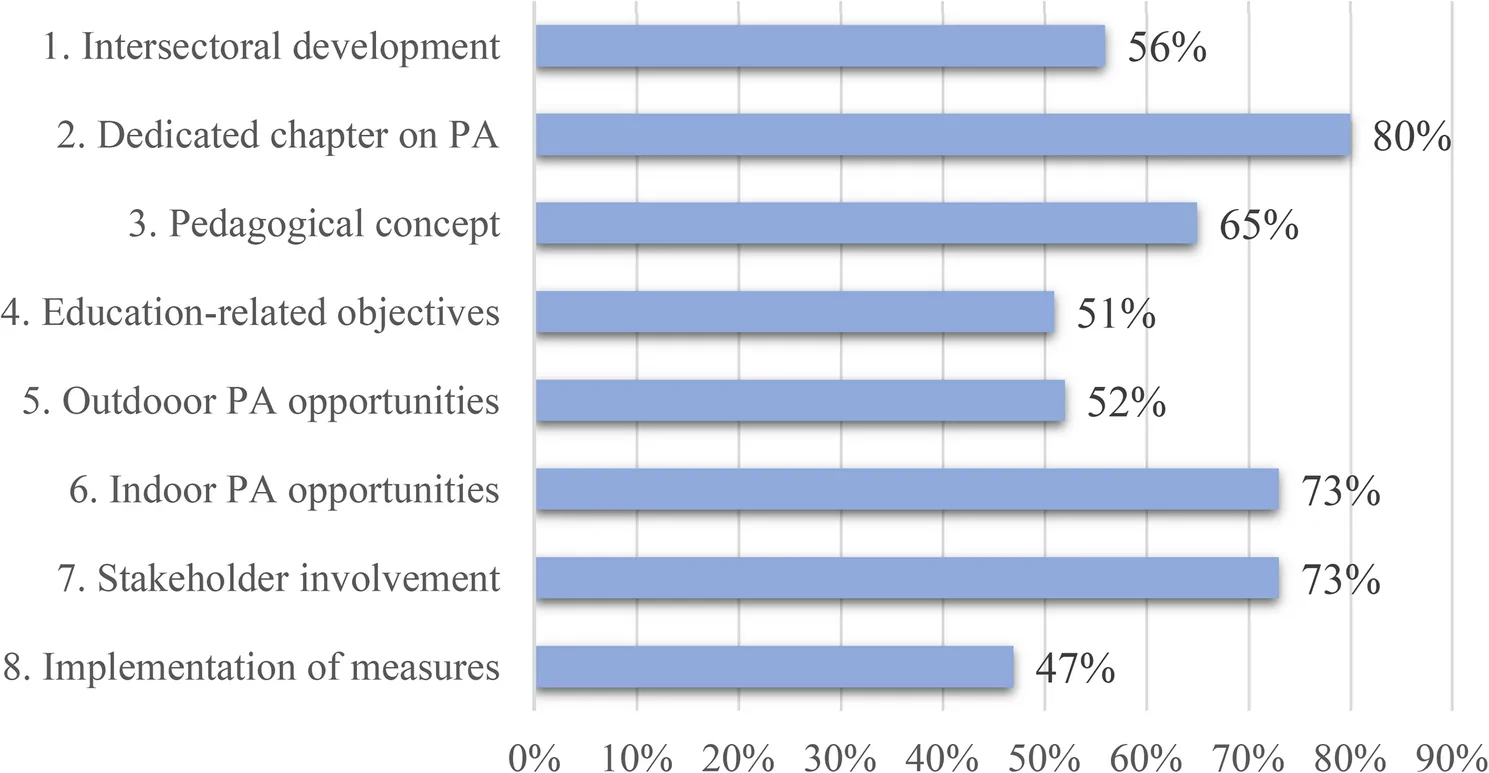
Results from the pilot test (*n* = 5), displayed as mean share of indicators met in each thematic domain

The pilot test displayed noticeable variance regarding the total number of fulfilled indicators. On average, 58% of all indicators were fulfilled across the five participating federal states (range: 46%–78%). Figure 2 displays the mean scores of the pilot test. 

All or most federal states involved state ministries for youth / family (100%) or education (80%) in the development of educational framework plans, while ministries for sport (40%), health (20%) and social affairs (20%) were rarely involved. With regards to stakeholder participation, consistent involvement was reported for pedagogical practice and scientific institutions (both 100%) and moderate involvement for parents (60%) and public bodies (80%), whereas sports associations (20%) and children (0%) were involved less often. A dedicated subchapter on PA was included in four out of five federal states (80%). Regarding a pedagogical concept, most federal states considered children’s individual needs (80%), whereas only one educational framework plan of the participating states referred to a scientifically grounded concept of PA or formulated a well-founded guiding principle (20%). All five educational framework plans emphasized the integration of PA into daily routines (100%) and its combination with other areas of education (100%). In contrast, mandatory education-related objectives were not reported in any state (0%), and only some states specified competencies (20%), included recommendations on regular guided PA sessions (40%) and on specific types of PA (60%), or provided guidance on recommended amounts of PA (20%). Considerable variance was also seen for indicators on the topic of PA in outdoor and indoor spaces. On the one hand, four educational framework plans included recommendations on fixed play equipment (80%) and three on the availability of portable play material (60%) and the ecological design of outdoor areas (60%). On the other hand, two addressed aesthetic design principles (40%), and one provided information on the minimum size of outdoor areas (20%). Similarly, only one framework plan specified minimum size requirements for indoor areas (20%) and recommendations for the regular review and adaptation of spatial and material PA-resources (20%), respectively. Recommendations on the flexible use of indoor spaces, however, were present in all five (100%) and the provision of PA-related material to promote different types of movement in four federal states (80%). Four educational framework plans also provided information on the involvement of parents (80%) and external providers (80%) in PA promotion, while the training and further education of pedagogical staff was addressed in three of the plans (60%). Finally, concrete guidance for the implementation of PA measures was provided in four framework plans (80%), and recommendations to promote the inclusion of children with disabilities or socially disadvantaged children were included in three federal states (60%). A complete list of aggregate scores for each indicator is presented in Appendix 1.

Qualitative feedback during the workshop confirmed the instrument’s applicability within federal health reporting. The five participants – one government official from each state – reported that the completion time varied depending on the length and complexity of the educational framework plans, but was generally considered appropriate, ranging from about one to six hours (including time to provide detailed feedback). In general, participants perceived the application of the instrument as straightforward and viewed its structure as clear and logical. Some feedback concerned potential overlaps between indicators, with suggestions being made to merge or streamline certain sections. Caution was expressed against further shortening, as this could reduce the clarity of individual indicators. Adjustments were made to improve comprehensibility, including a slightly revised instrument layout as well as adapted wording and refinement of single indicators. The manual was perceived as well-structured and of appropriate length, though several suggestions were made to improve its clarity and alignment with the instrument. The scoring system was considered intuitive, and it was suggested that domain-specific sub-scores would be more appropriate than an overall sum score, which would have required the different thematic domains to be weighted.

### Assessment instrument

The final version of the assessment instrument comprises eight thematic domains with a total of 28 indicators. While Table 1 provides an outline of its structure and lists abbreviated indicator descriptions, a full version can be found in Appendix 2.

**Table 1:** Overview of the assessment instrument

Domain		Corresponding indicators
1. Intersectoral development of educational framework plans	1.1	Involvement of state ministries in policy development (5 items).
	1.2	Involvement of other stakeholders in policy development (6 items).
2. Dedicated chapter on PA	2	Subchapter on PA in educational framework plan.
3. Pedagogical concept	3.1	Based on scientifically sound concept of the term ‘physical activity’.
	3.2	Considers the individual needs of children.
	3.3	Formulates a coherent and well-founded guiding principle for PA.
	3.4	Contains recommendations for further development of pedagogical concepts of ECEC centers regarding PA promotion.
4. Education-related objectives	4.1	Contains specific education-related objectives on PA.
	4.2	Includes recommendations for regular guided PA sessions.
	4.3	Includes recommendations for integrating PA promotion into daily routines in the ECEC setting.
	4.4	Emphasizes linking PA promotion with other areas of education.
	4.5	Specifies age- and development-appropriate competencies to be acquired through PA.
	4.6	Provides recommendations on specific amounts of PA.
	4.7	Provides recommendations on specific types of PA.
5. Outdoor PA opportunities	5.1	Includes recommendations for the flexible use of outdoor space for PA.
	5.2	Draws attention to the selection of fixed play equipment that is in line with the children’s development.
	5.3	Recommends the availability of portable play material.
	5.4	Includes recommendations for the ecological design of outdoor areas.
	5.5	Recommends designing outdoor areas according to aesthetic principles.
6. Indoor PA opportunities	6.1	Includes recommendations for the flexible use of different indoor spaces for PA.
	6.2	Recommends providing PA-related material to promote different types of movement.
	6.3	Includes recommendations for the adaptation of spatial and material resources to meet the children’s changing needs in regards to PA.
7. Stakeholder involvement	7.1	Draws attention to the involvement of parents in PA promotion.
	7.2	Contains suggestions for involving external providers in PA promotion.
	7.3	Encourages the training and further education of pedagogical staff in the field of PA.
8. Implementation of measures	8.1	Provides guidance for the implementation of PA measures.
	8.2	Includes recommendations for promoting the inclusion of children with disabilities in the context of PA.
	8.3	Includes recommendations for promoting the inclusion of socially disadvantaged children in the context of PA.

*PA* physical activity

## Discussion

To our knowledge, this is the first assessment instrument specifically designed to assess the PA-related content of public policies in the ECEC setting. The instrument focuses on the content of specific policy documents rather than assessing policy implementation or impact. It was developed through a structured, multi-phase co-production process involving researchers and political administration representatives from German health reporting departments and integrates evidence from policy document analyses, a systematic review, and recommendations on PA promotion in the ECEC setting. The initial set of indicators was revised, shortened, and adapted during a two-stage Delphi process, resulting in a final version comprising 28 indicators across eight thematic domains. A pilot test with representatives from five German federal states evaluated the instrument’s practical applicability and feasibility for use in health reporting.

From a national perspective, this instrument is innovative, as health monitoring systems in Germany have mainly included quantitative indicators (e.g., prevalence of health-related behaviors or health outcomes). This is a first attempt to assess PA-related policy content in educational framework plans. As government officials from the health sector evaluate the content of youth sector policies, this instrument may help to strengthen PA promotion in educational framework plans. Ultimately, this contributes to implementing the Health in All Policies (HiAP) approach in Germany, and complements recent policy recommendations of sport federations that call for strengthening PA promotion in the ECEC setting [49]. This national relevance aligns with long-standing international endeavors emphasizing the need for coordinated, cross-sectoral public health action. With the adoption of the *Ottawa Charter for Health Promotion* in 1986 [50], which first highlighted that improving population health requires coordinated action across sectors, the HiAP approach has become a central principle in international health promotion. To support broader uptake of this approach, both the *Helsinki Statement on HiAP* [51] and the *Adelaide Statement II* [52] have been adopted and reiterate that improving population health requires instruments and accountability mechanisms that allow sectors to systematically assess how their policies influence health and wellbeing. In this sense, the assessment instrument developed in this study provides a concrete mechanism to strengthen intersectoral collaboration, supporting the health sector in identifying opportunities to integrate PA-promoting content into youth sector policies.

In addition, the instrument was informed by normative considerations of what constitutes “good” policy. The Daugbjerg framework used for this study emphasizes that effective policy documents specify clear objectives, target groups, implementation mechanisms, responsibilities, timelines and, where possible, evaluation procedures. Similarly, the HARDWIRED criteria, proposed by Bellew et al. [53], outline key characteristics of successful PA policy processes, including highly consultative development, multi-level partnerships, adequate resourcing, synergy with other policy areas, wide communication, independent evaluation, clarified roles and responsibilities, and a foundation in evidence. Related work has highlighted the need to place PA firmly on the policy agenda through coherent, well-articulated and actionable policy instruments [54], further underlining the relevance of such criteria. While not all of these elements could be operationalized within the scope of this study, they provide a valuable reference point for future policy development.

This instrument can also be considered as an example for bridging the gap between scientific evidence and policy action through a co-production process. It includes indicators that were derived from the scientific literature [23, 37] and from an analysis of policy documents [38], ensuring that both perspectives are reflected. This guaranteed that the instrument reflects the PA-related content of educational framework plans, including aspects such as competence development (education-related objectives), structural environment (indoor and outdoor PA opportunities), and social environment (involvement of pedagogical staff, parents, and sport clubs). By incorporating real-world examples from educational framework plans, the instrument may also facilitate policy learning across federal states [55, 56]. In the context of this study, policy learning may take place when government officials compare the content of their own educational framework plan with those of other states, identify differences in policy design, and subsequently adjust their beliefs about what constitutes sufficiently detailed, evidence-informed, or ambitious PA promotion in the ECEC setting. In this way, the instrument provides a structured basis for reflection and cross-state learning that may inform future revisions of educational framework plans. Policy learning has further been described “as the updating of beliefs about policies resulting from the interaction of a wide range of policy actors involved in evaluation processes” [57], reflecting the relevance of the instrument’s collaborative development. Co-production processes have been shown to strengthen the relevance and practical uptake of policy instruments by integrating scientific knowledge with practitioner expertise and lived experience [32]. In this study, involving government officials from the health reporting departments ensured that the instrument was not only evidence-informed but also feasible to apply in an administrative context, thereby increasing the likelihood that it will be used and maintained over time. This co-production process also constitutes a form of *learning governance* [58], a conceptual approach in which policy actors deliberately shape and design learning processes – including who participates, underlaying knowledge, and how results are interpreted – to support both technical and political objectives. Through the use of evidence-informed indicators for structured assessment of PA policies in ECEC, the instrument may provide mechanisms for mutual learning between the health and youth sectors. This is also facilitated by integrating a pedagogical perspective on PA promotion in the assessment instrument, as the indicators on education-related objectives and competences relate to the priorities of the youth sector.

From an international perspective, this is the first policy assessment instrument specifically tailored to PA promotion in the ECEC sector. Currently, this perspective is only addressed by a single indicator in WHO’s GAPPA Monitoring Framework [11]. In the first round of data collection, only 30% of countries reported implementing national policies promoting PA in child-care settings [27]. Consequently, this instrument may be most useful for countries that already have a policy in place – but may also inspire or guide countries that aim at developing such a policy. In this context, users of this instrument may also consider that PA promoting policies in the ECEC setting are often vaguely worded, lack alignment with WHO 2019 guidelines, and are rarely informed by robust evidence [59]. This is particularly important, as this instrument aims to assess policy content (rather than policy implementation or impact). Insights from the development of other PA policy monitoring instruments may also be relevant for this study. Similar to our instrument, the MOVING tool aims to assess the quality of policy design against evidence-based, aspirational standards [60]. Such aspirational standards set a higher bar for governments than comparing policies to current best practice, and have the potential to encourage policy innovation. While the MOVING benchmarking tool is designed to collect data across multiple sectors, our instrument focuses specifically on the ECEC setting. However, both instruments allow the structured comparison of policy content across countries or subnational units of government by defining a clear set of desired policy attributes that allows the identification of gaps.

Future research may investigate the use of the instrument in Germany, e.g., whether assessments inform future decision-making processes and how benchmarking is perceived by government officials in the youth sector. In addition, the experiences collected during this project may help to operationalize other policy indicators for the prevention indicator system of the German federal states (e.g., “health literacy is considered in the educational framework plans of ECEC and schools”). Furthermore, it needs to be explored whether this instrument is transferable to other countries: While its original purpose is to assess state-level ECEC policies in Germany – where responsibility for ECEC policy lies largely with the youth sector at subnational level – in other countries, comparable responsibilities may be located at other levels of government. Consequently, the suitability and relevance of individual indicators within different administrative contexts needs to be examined.

Several limitations of this study need to be acknowledged. First, the study was informed by a content analysis of ECEC policies in Germany, i.e. the political context which differs between countries. Second, even though the standardized use of the instrument is supported by a manual, some degree of interpretation by users remains inevitable. Third, while the instrument allocates points at the level of thematic domains, it does not apply an explicit weighting of individual indicators. This may be viewed as a limitation, particularly because some aspects may be more relevant for PA promotion than others. Fourth, the instrument does not cover all criteria on ‘good policies’, as it was designed to assess aspects that are typically addressed within educational framework plans. In particular, the instrument does not assess evaluation mechanisms, timeframes, or budget specifications. Finally, the instrument was developed to assess policy content rather than policy implementation or policy impact in the ECEC setting.

## Conclusions

This study developed the first instrument specifically designed to assess the PA–related content of ECEC policies, comprising 28 indicators across eight domains. As the instrument was found to be feasible and usable by state‑level health reporting authorities, it may help to strengthen the integration of PA promotion within early childhood policy. Despite being developed within the German context, it may be useful to inform similar monitoring efforts in other countries and guide future research on PA promotion within the ECEC setting.

## Supplementary Information

Supplementary Material 1.

## Data Availability

The data and materials used and/or analysed during the current study are available from the corresponding author on reasonable request. The assessment instrument and the complementary manual are also available in an online repository (https://doi.org/10.17605/OSF.IO/3CJXF).

## Abbreviations

PA: Physical activity
ECEC: Early childhood education and care
WHO: World Health Organization
GAPPA: Global Action Plan on Physical Activity
