# Supplementary material for: Co-producing an assessment instrument for physical activity policies in the early childhood education and care setting
Source: Int J Behav Nutr Phys Act. 2026 Oct 22;23:102. doi: 10.1186/s12966-026-02001-z (PMC13700408; doi:10.1186/s12966-026-02001-z)
Supplement: Supplementary file 1 [file 12966_2026_2001_MOESM1_ESM.odt]

Appendix

Appendix 1. Aggregate number of pilot participants answering an indicator with "yes"
No.
Indicator
Aggregate score
1.1
Representatives from the following state ministries were involved in the development of the educational framework plan: Education
4/5 (80%)

Representatives from the following state ministries were involved in the development of the educational framework plan: Health
1/5 (20%)

Representatives from the following state ministries were involved in the development of the educational framework plan: Youth / Family
5/5 (100%)

Representatives from the following state ministries were involved in the development of the educational framework plan: Sport
2/5 (40%)

Representatives from the following state ministries were involved in the development of the educational framework plan: Social Affairs
1/5 (20%)
1.2
The following stakeholders were involved in the development of the educational framework plan: Pedagogical / educational practice
5/5 (100%)

The following stakeholders were involved in the development of the educational framework plan: Children
0/5 (0%)

The following stakeholders were involved in the development of the educational framework plan: Parents (or parents’ associations)
3/5 (60%)

The following stakeholders were involved in the development of the educational framework plan: Public bodies (public-municipal providers, welfare associations / churches)
4/5 (80%)

The following stakeholders were involved in the development of the educational framework plan: Sports associations
1/5 (20%)

The following stakeholders were involved in the development of the educational framework plan: Scientific institutions / academia
5/5 (100%)
2
The educational framework plan includes a subchapter on the topic of physical activity.
4/5 (80%)
3.1
The educational framework plan is based on a scientifically sound concept of the term 'physical activity' (e.g., considering motor, social, and cognitive dimensions of development).
1/5 (20%)
3.2
The educational framework plan takes into account the individual needs of children (e.g., by involving them in the design and implementation of physical activity opportunities).
4/5 (80%)
3.3
The educational framework plan formulates a coherent and well-founded guiding principle regarding physical activity.
1/5 (20%)
3.4
The educational framework plan contains concrete recommendations for the regular reflection and further development of the pedagogical concept of ECEC centers regarding physical activity promotion.
2/5 (40%)
4.1
The educational framework plan contains mandatory education-related objectives on the topic of physical activity.
After the pilot test, the indicator was changed to “The educational framework plan contains specific education-related objectives on the topic of physical activity”.
0/5 (0%)
4.2
The educational framework plan includes recommendations for regular guided physical activity sessions (e.g., by providing specific examples and opportunities for implementing structured PA).
2/5 (40%)
4.3
The educational framework plan includes recommendations for integrating physical activity promotion into daily routines in the ECEC setting, laying emphasis on unstructured or non-guided physical activity.
5/5 (100%)
4.4
The educational framework plan emphasizes linking physical activity promotion with other areas of education (e.g., in relation to children’s social, cognitive, linguistic, or emotional development).
5/5 (100%)
4.5
The educational framework plan specifies age- and development-appropriate competencies to be acquired through physical activity (e.g., regarding one’s self-competence, knowledge competence, social competence, and learning competence).
2/5 (20%)
4.6
The educational framework plan provides recommendations on specific amounts of physical activity (duration, frequency, regularity, intensity).
1/5 (20%)
4.7
The educational framework plan provides recommendations on specific types of physical activity (e.g., grabbing, throwing, crawling, running, jumping, or climbing).
3/5 (60%)

The educational framework plan includes information on the minimum size of outdoor areas.
After the pilot test, the indicator was changed to “The educational framework plan includes recommendations for the flexible use of outdoor space for physical activity”.
1/5 (20%)
5.2
The educational framework plan draws attention to the selection of fixed play equipment that is in line with the children’s development (e.g., swings, slides, seesaws).
4/5 (80%)
5.3
The educational framework plan recommends the availability of portable play material (e.g., balls, rackets, frisbees).
3/5 (60%)
5.4
The educational framework plan includes recommendations for the ecological design of outdoor areas.
3/5 (60%)
5.5
The educational framework plan recommends designing outdoor areas according to aesthetic principles (based on a jointly constructed aesthetic perception of both children and educational staff).
2/5 (40%)

The educational framework plan includes information on the minimum size of outdoor areas.
The indicator was removed after the pilot test.
1/5 (20%)
6.1
The educational framework plan includes recommendations for the flexible use of different indoor spaces for physical activity (e.g., group room, activity room, hallway area).
5/5 (100%)
6.2
The educational framework plan recommends providing PA-related material to promote different types of movement (e.g., climbing frames, material for building and construction, portable equipment such as balls and hoops).
4/5 (80%)
6.3
The educational framework plan includes recommendations for the regular review and adaptation of spatial and material resources to meet the children’s changing needs in regards to physical activity.
1/5 (20%)
7.1
The educational framework plan draws attention to the involvement of parents in physical activity promotion (by providing specific suggestions and examples for implementation, or references to relevant educational offers, such as parent conferences with experts).
4/5 (80%)
7.2
The educational framework plan contains suggestions for involving external providers in physical activity promotion (e.g., through cooperation with sports clubs or primary schools).
4/5 (80%)
7.3
The educational framework plan encourages the training further education of pedagogical staff in the field of physical activity (e.g., regarding the implementation of physical activity opportunities and the development of a role model function).
3/5 (60%)
8.1
The educational framework plan provides concrete guidance for the implementation of physical activity measures (practical examples or suggestions for ECEC centers, such as “Forest Days” or movement-related games).
4/5 (80%)

The educational framework plan includes recommendations or implementation examples for promoting the inclusion of children with disabilities or socially disadvantaged children in the context of physical activity.
After the pilot test, the indicator was split into two individual indicators, distinguishing between “recommendations or implementation examples for promoting the inclusion of children with disabilities” and “recommendations or implementation examples for promoting the inclusion of socially disadvantaged children.
3/5 (60%)


Appendix 2. Complete version of the assessment instrument
Domain

Corresponding indicators
1. Intersectoral development of educational framework plans
1.1
Representatives from the following state ministries were involved in the development of the educational framework plan:
Education
Health
Youth / Family
Sport
Social Affairs

1.2
The following stakeholders were involved in the development of the educational framework plan:
Pedagogical / educational practice
Children
Parents (or parents’ associations)
Public bodies (public-municipal providers, welfare associations / churches)
Sports associations
Scientific institutions / academia
2. Dedicated chapter on PA
2
The educational framework plan includes a subchapter on the topic of physical activity.
3. Pedagogical concept
3.1
The educational framework plan is based on a scientifically sound concept of the term 'physical activity' (e.g., considering motor, social, and cognitive dimensions of development).

3.2
The educational framework plan takes into account the individual needs of children (e.g., by involving them in the design and implementation of physical activity opportunities).

3.3
The educational framework plan formulates a coherent and well-founded guiding principle regarding physical activity.

3.4
The educational framework plan contains concrete recommendations for the regular reflection and further development of the pedagogical concept of ECEC centers regarding physical activity promotion.
4. Education-related objectives
4.14.1
The educational framework plan contains specific education-related objectives on the topic of physical activity.

4.2
The educational framework plan includes recommendations for regular guided physical activity sessions (e.g., by providing specific examples and opportunities for implementing structured PA).

4.3
The educational framework plan includes recommendations for integrating physical activity promotion into daily routines in the ECEC setting, laying emphasis on unstructured or non-guided physical activity.

4.4
The educational framework plan emphasizes linking physical activity promotion with other areas of education (e.g., in relation to children’s social, cognitive, linguistic, or emotional development).

4.5
The educational framework plan specifies age- and development-appropriate competencies to be acquired through physical activity (e.g., regarding one’s self-competence, knowledge competence, social competence, and learning competence).

4.6
The educational framework plan provides recommendations on specific amounts of physical activity (duration, frequency, regularity, intensity).

4.7
The educational framework plan provides recommendations on specific types of physical activity (e.g., grabbing, throwing, crawling, running, jumping, or climbing).
5. Outdoor PA opportunities
5.15.1
The educational framework plan includes recommendations for the flexible use of outdoor space for physical activity.

5.2
The educational framework plan draws attention to the selection of fixed play equipment that is in line with the children’s development (e.g., swings, slides, seesaws).

5.3
The educational framework plan recommends the availability of portable play material (e.g., balls, rackets, frisbees).

5.4
The educational framework plan includes recommendations for the ecological design of outdoor areas.

5.5
The educational framework plan recommends designing outdoor areas according to aesthetic principles (based on a jointly constructed aesthetic perception of both children and educational staff).
6. Indoor PA opportunities
6.16.1
The educational framework plan includes recommendations for the flexible use of different indoor spaces for physical activity (e.g., group room, activity room, hallway area).

6.2
The educational framework plan contains suggestions for involving external providers in physical activity promotion (e.g., through cooperation with sports clubs or primary schools).

6.3
The educational framework plan includes recommendations for the regular review and adaptation of spatial and material resources to meet the children’s changing needs in regards to physical activity.
7. Stakeholder involvement
7.17.1
The educational framework plan draws attention to the involvement of parents in physical activity promotion (by providing specific suggestions and examples for implementation, or references to relevant educational offers, such as parent conferences with experts).

7.2
The educational framework plan contains suggestions for involving external providers in physical activity promotion (e.g., through cooperation with sports clubs or primary schools).

7.3
The educational framework plan encourages the training further education of pedagogical staff in the field of physical activity (e.g., regarding the implementation of physical activity opportunities and the development of a role model function).
8. Implementation of measures
8.18.1
The educational framework plan provides concrete guidance for the implementation of physical activity measures (practical examples or suggestions for ECEC centers, such as “Forest Days” or movement-related games).

8.2
The educational framework plan includes recommendations or implementation examples for promoting the inclusion of children with disabilities in the context of physical activity.

8.3
The educational framework plan includes recommendations or implementation examples for promoting the inclusion of socially disadvantaged children in the context of physical activity.
